# Stronger responders—uptake and decline of HPV-vaccination in Denmark

**DOI:** 10.1093/eurpub/cky235

**Published:** 2018-11-08

**Authors:** Charlotte Lynderup Lübker, Elsebeth Lynge

**Affiliations:** Centre for Epidemiological Research, Nykøbing Falster Hospital, University of Copenhagen, Copenhagen, Denmark

## Abstract

**Background:**

The purpose of this study was to identify the stronger responders behind the fluctuating coverage with the HPV-vaccine in Denmark in order to facilitate information campaigns targeted at specific subgroups.

**Methods:**

Newspaper articles published from 2006 to 2009 with information about coverage with the HPV-vaccine in Denmark were identified from the database Infomedia.dk. Vaccination coverage of recent years was retrieved from the publically accessible statistics from the State’s Serum Institute. Data on average disposable income nationally and for each municipality was retrieved from Statistics Denmark.

**Results:**

According to numbers published in newspapers, girls residing in municipalities with a high disposable income were the first ones to secure the HPV-vaccine in Denmark. Years later, at the start of the debate about possible side effects of the HPV-vaccine, the decline in vaccination coverage was slightly steeper for girls from high income municipalities than for girls from low income municipalities.

**Conclusions:**

Girls from municipalities with a high disposable income seem to be the stronger responders of the fluctuating coverage with the HPV-vaccine in Denmark. This was the case both during the initial surge in coverage after the vaccine’s introduction on the market, and during the later decline following the debate on possible side effects. Identification of this dispersion pattern enables health authorities to initiate targeted information campaigns.

## Introduction

The quadrivalent vaccine Gardasil^®^ against human papillomavirus (HPV) types 6, 11, 16 and 18 was introduced in Denmark in October 2006.^1^ Whilst offering protection against the virus types most commonly causing condylomas and cervical dysplasia,^2^^,^^3^ it came at a price of 470 euros for the three recommended doses.^4^ In October 2008, the HPV-vaccine was introduced into the Danish Childhood Vaccination Programme at the expense of the state, making the vaccine free of charge for the citizens. From January 2009, girls born in 1996 and later were offered the vaccine free of charge from the age of 12–18. A catch-up programme was installed running from October 2008 to December 2010, offering girls born from 1993 to 1995 the vaccine free of charge, whilst another catch-up programme running from September 2012 to December 2013 covered women born from 1985 to 1992. This inclusion into the Childhood Vaccination Programme was a popular move as the vaccine was expensive and the risk of developing cervical cancer is highest in the lowest socioeconomic groups.^5^^–^^7^ Furthermore, Denmark has a high background risk of cervical cancer compared to the rest of Western Europe,^8^ and a suboptimal participation in the Cervical Cancer Screening Programme.^9^ The introduction of the HPV-vaccine was thus a promising new step towards combatting cervical cancer in Denmark.

Data from the national vaccination register show that after the introduction of the HPV-vaccination programme, the first dose coverage for girls born in 1997–2000 was constant at around 90%, and full immunization coverage was around 80%.^10^^,^^11^ However, a decline in coverage started in 2013, first registered for girls born in 2001, and full immunization coverage reached a mere 15% for girls born in 2004.^11^ The decline in coverage followed worries in the lay press and on social media about possible side effects of HPV-vaccination.^12^^,^^13^

We sought to identify the stronger responders in the HPV-vaccine’s fluctuating uptake in Denmark. First, we searched for data on stronger responders in the uptake of the HPV-vaccination between the marketing of the vaccine in September 2006, and the inclusion of the vaccine into the Danish Childhood Vaccination Programme in October 2008. Subsequently, we investigated the stronger responders to discard the vaccine from 2012 onwards, focussing in particular on girls born in 2000–04. Responders were characterized by geographical distribution and income in order to determine whether information on the HPV-vaccine should be tailored to particular population subgroups.

## Methods

Newspaper articles were examined to collect data on stronger responders to accept the HPV-vaccine. A search was made on the database Infomedia.dk for all newspaper articles containing the words ‘HPV vaccine’ between 1 September 2006 and 1 September 2009. The term HPV-vaccine was well known in the Danish public, and it is unlikely that a newspaper article covering this topic would not have included this term. Infomedia is a comprehensive Danish data base including all daily nationwide, regional and local as well as weekly newspapers. The search was made directly on the Infomedia website by one of the authors (C.L.L.). University students can make search on Infomedia free of costs. The search revealed 1182 articles. The title and the first 20–25 words of each article were read. As newspaper articles start with presenting the ‘results’ before ‘material and methods’, the relevance of the content can easily be identified from the first words. The articles expected to provide information on number of and/or characteristics of HPV-vaccinated girls and young women were selected. One of the authors (C.L.L.) selected the relevant newspaper articles. Normally, there are more than one copy of a given article, e.g. from a nationwide newspaper and from several local newspapers. The risk of overlooking a given article is therefore small. If two or more newspaper articles contained identical data, only one was selected and used in the results. Information of date, source, municipality/region, age group if provided and reported purchase/use of HPV-vaccine was abstracted by one author (C.L.L.), and checked by the other author (E.L.).

The website of the State’s Serum Institute (SSI) was searched for data on first dose and full immunization of the HPV-vaccine for girls born in 2000, 2001, 2002, 2003 and 2004 nationwide and by municipality.^10^^,^^11^ These data were retrieved as percentages only. Data, updated on the 8 June 2017, were retrieved on 3 July 2017. Data from municipalities with <100 girls in one birth cohort were excluded due to small numbers. This meant exclusion of the municipalities of Dragør, Fanø, Langeland, Læsø, Samsø, Vallensbæk and Ærø. Data from the remaining municipalities were used to identify the stronger responders to discard the HPV-vaccine.

The website of Statistics Denmark was searched for data on average disposable income nationwide and by municipality.^14^ Data from years 2006 to 2009 and 2015 were retrieved on the 4 July 2017. The numbers retrieved were rounded to the nearest thousand Danish Krone (DKK).

## Results

### Stronger responders to secure the HPV-vaccine

A newspaper article published on 8 December 2006 with data from the Danish Drug Information showed that while 33% of the HPV-vaccine doses were sold in the rich municipalities of North Zealand, not a single dose had been sold in Ishøj, with an average disposable income of 161 thousand DKK, falling beneath the national average of 173 thousand DKK (table 1 and Supplementary material). This trend was seen in all data from articles published in 2007 and 2008. According to a newspaper article from 22 November 2008 (table 1 and Supplementary Material) in the municipality of Hørsholm, with an average income far above the national average, 3000 vaccine doses were sold per 100 000 citizens in 2007 as compared with just over 200 doses per 10 000 citizens in the municipality of Lolland, with the lowest average income. According to a newspaper article from 3 May 2009, one-third of the eligible young women were reported to have bought the vaccine in Hørsholm, as opposed to the mere 1/50 of eligible young women in the municipalities of Høje Taastrup and Faaborg, with average incomes around and far below the national average, respectively. In 2009, a newspaper published Sanofi Pasteur’s data on vaccine coverage for women aged 16–26 years for several municipalities (table 2), and the 33.1% coverage in Hørsholm evidently contrasted the 1.4% coverage in Høje Taastrup. Furthermore, the three municipalities with the highest coverage had average disposable incomes clearly exceeding the national average of 179 thousand DKK, with Gentofte’s 275 thousand DKK topping the list.

**Table 1:** Data extracted from newspaper articles from 2006 to 2009 on stronger responders to secure the HPV-vaccine. Average disposable incomes in the municipalities included (Appendix A1)

DD/MM/YY	Source	Total vaccinated	Municipality	Average income in municipality^a^	Use of HPV-vaccine in municipality	Reference
08/12/06	Danish drug information	NA	Gentofte	300	33% of doses sold	(Appendix A2)
			Holte^b^	285		
			Nærum^b^	285		
			Vedbæk^b^	285		
			Ishøj	161	0	
08/02/07	Sanofi Pasteur	4300	Gentofte	302	ca. 50% of doses sold	(Appendix A3)
			Lyngby-Tårbæk	231		
			Rudersdal	287		
			Rødovre	176	ca. 0	
			Brøndby	166		
			Albertslund	165		
02/05/07	According to the minister	NA	North of Copenhagen^d^	231–302	Most doses sold here	(Appendix A4)
			Albertslund	165	ca. 0	
03/05/07	Sanofi Pasteur	NA	Gentofte	302	Doses sold in these municipalities	(Appendix A5)
			Lyngby-Tårbæk	231		
			Rudersdal	287		
			Brøndby	166	ca. 0	
			Albertslund	165		
09/08/07	NA	12 000	NA	NA	NA	(Appendix A6)
10/08/07	Sanofi Pasteur	NA	North Zealand^d^	231–302	Many girls vaccinated	(Appendix A7)
			Rødovre	176	Only a few girls vaccinated	
			Albertslund	165		
			Brøndby	166		
02/11/07	Sanofi Pasteur	NA	Rudersdal	287	1057/2351^e^ women aged 16–26 vaccinated	(Appendix A8)
			Helsingør	198	357/2807^e^ women aged 16–26 vaccinated	
			Albertslund	165	57/2007^e^ women aged 16–26 vaccinated	
09/11/07	Sanofi Pasteur	NA	Furesø	227	386 vaccinated, total 1663 women aged 16–26^e^	(Appendix A9)
			Rudersdal	287	1057 vaccinated, total 2351 women aged 16–26^e^	
			Høje Taastrup^c^	180	71 vaccinated, total 2847 women aged 16–26^e^	
			Albertslund	165	57 vaccinated, total 2007 women aged 16–26^e^	
12/11/07	Sanofi Pasteur	11 500	Rich municipalities of North Zealand^d^	231–302	Many more vaccinated	(Appendix A10)
21/11/07	NA	NA	Hørsholm	291	ca. 30% aged 16–26 vaccinated	(Appendix A11)
			Rudersdal	287	ca. 30% aged 16–26 vaccinated	
			Høje Taastrup	180^c^	3–6% aged 16–26 vaccinated	
			Albertslund	165		
20/12/07	NA	NA	Rudersdal	287	ca. 2000 vaccinated/100 000 inhabitants	(Appendix A12)
			Vejle	180^c^	180 vaccinated/100 000 inhabitants	
			Horsens	171	69 vaccinated/100 000 inhabitants	
30/01/08	NA	21 000	NA	NA	NA	(Appendix A13)
22/11/08	Sanofi Pasteur	NA	Hørsholm	260	2980 doses sold/100 000 inhabitants	(Appendix A14)
			Lolland	151	218 doses sold/100 000 inhabitants	
03/05/09	NA	NA	Hørsholm	251	1/3 aged 17–26 have bought vaccine	(Appendix A15)
			Høje Taastrup	184^c^	1/50 aged 17–26 have bought vaccine	
			Faaborg	167		
28/05/09	Sanofi Pasteur	NA	Århus	178	ca. 15% aged 16–26 vaccinated	(Appendix A16)

All quotes translated from Danish to English.

^a^Disposable income in the given year of the newspaper article, rounded to the nearest thousand DKK. National average disposable incomes in thousands of DKK in the given years: 2006 [173], 2007 [178], 2008 [176] and 2009 [179] (Appendix A1).

^b^These three municipalities were joined in one municipality called Rudersdal as part of a reform in 2007. On Statistics Denmark one can only select the names of the new municipalities, therefore the disposable income of Rudersdal is used to represent the disposable income in these former three municipalities.

^c^Average disposable income marginally above national average.

^d^North of Copenhagen and North Zealand include the municipalities listed by names as high income municipalities.

^e^Supplementary data on number of women aged 16–26 in each municipality added to data from newspaper article.

[*ref: BEF1A07: Folketal 1. januar efter kommune, køn, alder og civilstand (afsluttet): Danmarks Statistik; (cited 2018 Sep 30). Available from: **https://www.statistikbanken.dk/BEF1A07*.].

**Table 2:** Data from newspaper in 2009, showing the three municipalities with the highest and lowest coverage of HPV-vaccinated girls/women aged 16–26 years (Appendix A17). Included average disposable income in the municipalities (Appendix A1)

Municipalities	Average disposable income 2009^a^ (thousands DKK)	Fully vaccinated or being vaccinated (%)
Highest coverage		
Hørsholm	251	33.1
Gentofte	275	21.9
Rudersdal	255	21.9
Lowest coverage		
Faxe	176	2.5
Faaborg	167	2.0
Høje Taastrup	184	1.4

^a^Average disposable income in 2009 was 179 thousand DKK (Appendix A1).

### Stronger responders to discard the HPV-vaccine

**Figure 1:**
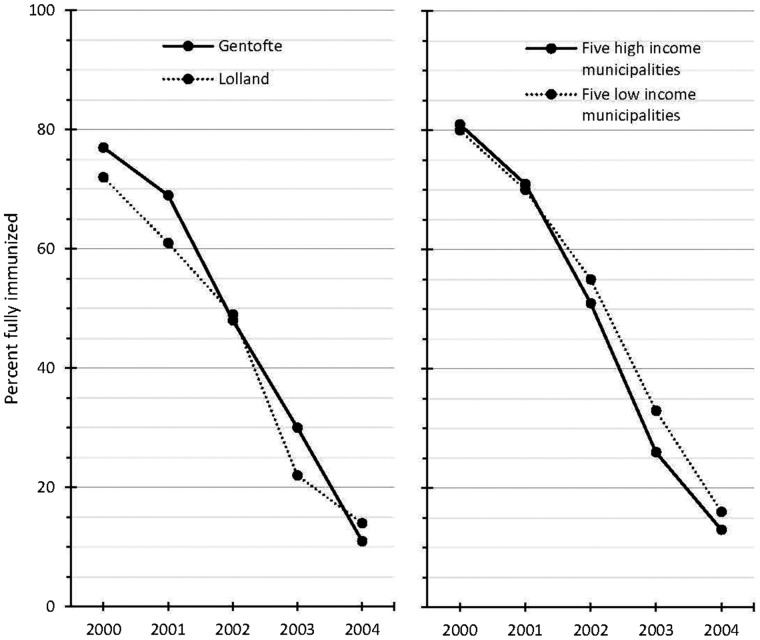
Percent of girls fully immunized with HPV-vaccine by year of birth

Gentofte, being the municipality with the highest average income, saw a dramatic decrease in the percentage of fully immunized girls from 77% for girls born in 2000—69% for girls born in 2001, and further decreasing to 48, 30 and 11%, respectively, for girls born in 2002, 2003 and 2004 (table 3 and figure 1) A decline was seen also for girls in Lolland, having the lowest average disposable income, where full immunization went from 72% for girls born in 2000, to 61% for 2001, 49% for 2002, 22% for 2003 and14% for 2004. The decline was slightly more marked in Gentofte than in Lolland, overall 86% [= (77−11)/77] in Gentofte and 81% [= (72−14)/72] in Lolland. The five municipalities with the highest income and the five municipalities with the lowest income started at almost the same level of full immunization for girls born in 2000; 81 and 80%, respectively. But also here the decline was slightly more marked for the high income municipalities; 84% [=(81–13)/81)], than for the low income municipalities; 80% [=(80–16)/80)]. Although the coverage for first vaccine dose was in general higher, similar declining trends were seen. In Gentofte, the coverage declined from 91% for girls born 2000—37% for girls born in 2004, whilst the decline in Lolland was slightly more modest from 90% for girls born in 2000—42% for girls born in 2004 (table 3). All girls born between 2000 and 2004 had been offered HPV-vaccination from the age of 12. The oldest of these cohorts born in 2000 turned 12 years in 2012, before the circulation of worries about possible side-effect of HPV-vaccination, while the youngest of these cohorts born in 2004 turned 12 years in 2016 in the mist of the storm.

**Table 3:** The five richest and five poorest municipalities with average disposable income (Appendix A1) and percentage of girls fully immunized and girls with first dose of HPV-vaccine by year of birth, 2000–04 (Appendixes A18 and A19)

Municipality	Average income 2015 (thousand DKK)	Full immunization Vaccination coverage (%)					First vaccine dose Vaccination coverage (%)				
		2000	2001	2002	2003	2004	2000	2001	2002	2003	2004
Age at data retrieval in years		16–17	15–16	14–15	13–14	12–13	16–17	15–16	14–15	13–14	12–13
Highest income											
Gentofte	425	77	69	48	30	11	91	87	77	56	37
Rudersdal	379	79	67	50	22	10	93	85	79	48	34
Hørsholm	355	83	67	49	18	15	94	91	80	46	40
Lyngby-Taarbæk	322	83	76	51	30	11	94	90	78	57	42
Allerød	288	82	75	56	32	17	96	92	86	64	48
Average	354	81	71	51	26	13	94	89	80	54	40
Lowest income											
Lolland	185	72	61	49	22	14	90	84	80	44	42
Bornholm	189	85	74	55	34	14	95	87	79	52	40
Tønder	190	80	66	60	33	15	92	80	88	55	45
Morsø	191	79	75	55	40	15	90	93	83	62	38
Vesthimmerland	193	82	72	55	35	22	90	90	82	55	42
Average	190	80	70	55	33	16	91	87	82	54	41

## Discussion

This study revealed that girls living in municipalities with a high average disposable income appeared to be stronger responders of the varying coverage of the HPV-vaccine in Denmark. They were the first to secure the HPV-vaccine after it was marketed, and they had the steepest decline in vaccination coverage during the debate about possible side effects of the HPV-vaccine.

This trend, that the richer parts of society are the stronger responders in terms of vaccination, is not a new phenomenon. Looking back at the measles, mumps and rubella (MMR) vaccine scare, similar tendencies were observed. Studies from the UK indicated that during the scare about the MMR vaccine, the largest decrease in vaccination participation was seen in wealthy or well-educated areas.^15^^,^^16^ According to one of the studies, the major part of the decline was seen during the early stages of the controversy with the people from higher social classes having the fastest decline.^15^ A similar pattern was evident in our results, where the decline in coverage was slightly steeper in the wealthiest than in the poorest municipalities. One may wonder whether this difference is due to obvious factors such as the wealthier part of the population having better access to information, better pre-requisite for interpreting information, and therefore able to react faster on new information, irrespective of the reliability of the source. This was investigated in several studies, with many results highlighting that people from higher social classes want to be given the choice, be well informed, and be regarded as competent to make health decisions.^17^^,^^18^ Concurrently, one study called attention to the fact that whilst rigorous research on the effect and safety of the vaccine is a requirement, research is lacking on factors which affect public trust in vaccines.^19^

Concerning the recent discussion of side effects to the HPV-vaccine in Denmark, a study from 1997 found that people tend to let information with high-risk indication militate more than information on low risk.^20^ This may have played a part in the rapidly declining participation in the HPV-vaccination progamme in Denmark, where the media have been responsible for many headlines concerning severe side effects of the vaccine without presenting any clear evidence. The few press releases from the government about the safety of the HPV-vaccine have seemingly simultaneously been neglected and outweighed. This power of the media is not unheard of. Previous studies suggest that the media coverage was the reason for a dramatic increase in influenza vaccinations in the USA,^21^ whilst the media portrayal of possible side effects to the pertussis vaccine in 1974 led to a decline in vaccination participation leading to two major epidemics of pertussis in the UK.^22^^,^^23^ The media’s unsubstantiated statements on risks are reinforced when they are disseminated on social media.

The study has a number of limitations. Firstly, data on stronger responders to secure the HPV-vaccine was based on numbers reported in newspaper articles and most of these numbers came from Sanofi Pasteur; the company marketing the Gardasil^®^ vaccine in Denmark. One has to be aware that it could have been in their interest to publish numbers which portrayed the social inequity in HPV-vaccination in order to put pressure on the government to include the HPV-vaccine in the Danish Childhood Vaccination Programme. It is important to point out that the company had this information reported in the standard news sections of the newspapers and not in advertizements. Secondly, numbers from the SSI provided a snapshot of the coverage at a specific point in time and did therefore not show the constant dynamics of the vaccine coverage. We realize that the data illustrate a tendency but do not provide a formal test of a hypothesis. Thirdly, as these number were retrieved as percentages only significance testing of differences was not possible. Lastly, the average disposable income in the municipality was used to characterize all inhabitants in this geographic area. However, the SSI also presents data on coverage by municipality, which means that the two data sets are comparable.

This study portrayed the rather unexpected turn in the coverage of the HPV-vaccination in Denmark, and it uncovered a common characteristic of the stronger responders. In other words, it seems that the girls living in a municipality with a high average disposable income were trendsetters on the HPV-vaccine coverage in Denmark. This common factor could be used in future government vaccination campaigns, where a possibility would be to target information more directly at this group of trendsetters with the hope that they would spread evidence-based information about the vaccine. This however inevitably raises ethical questions on social discrimination especially when it comes to a national vaccination programme, which is seen as a basic right regardless of socioeconomic class.

This study also fostered new inquiries on the mechanism of losing public confidence in a nationwide vaccination programme, to such a severe extent as with the HPV-vaccine in Denmark, and the question remains on how to restore public trust in the vaccine. A previous study suggested that once the MMR scare was over, the wealthier people were the first to resume use of the vaccination programme,^15^ whilst several other studies of attitudes towards vaccination indicated that the family doctor played a key role when parents decided on whether or not to vaccinate their children.^21^^,^^24^^–^^26^ Thus, focussing vaccination campaigns more locally at the family doctor, ensuring that they are prepared to give the girls and their parents neutral and evidence-based information, and combining it with information specifically targeting girls in municipalities with high average disposable income may be part of the solution to a vaccination crisis as the one seen with the HPV-vaccine in Denmark.

After massive national efforts to ensure equal access to the HPV-vaccine in Denmark, the vaccination coverage has recently seen a dramatic decline due to the public debate about possible side effects. To regain public trust and target vaccination campaigns, it is necessary to unveil the dynamics of these variations in vaccination coverage. This study indicated that girls residing in municipalities with a high average disposable income were the stronger responders of the fluctuation in coverage with HPV-vaccine in Denmark. In other words, according to our results they appeared to be both the first to opt into the newly approved vaccine, when it hit the Danish market, and later they seem somewhat faster to opt out of the vaccination programme, when the debate about possible side effects bloomed. If this is a continuous trend then this subgroup of the population may be crucial, when targeting campaigns to ensure high coverage in national vaccination programmes.

## Funding

This work was supported by Kirsten and Freddy Johansen’s Fund. It had no involvement in the study design, the collection, analysis and interpretation of data, the writing of the report or the decision to submit the paper.

## Disclaimer

Elsebeth Lynge: Roche provides test kits free of charge for a randomized controlled trial. Participated in meetings with Roche with fees paid to the University of Copenhagen. Has participated in one meeting organized by the HPV Prevention and Control Board and in one meeting organized by GSK; both without payment.

*Conflicts of interest*: None declared.

**Key points**

- In Denmark, girls from high income municipalities were the first to use the HPV vaccine after it was marketed in 2006.
- After the HPV-vaccine was included in the Danish Childhood Vaccination Programme in 2008, vaccine uptake become high throughout Denmark.
- A decline in HPV-vaccine uptake started in 2013 following worries in the lay press and social media about possible side effects.
- This decline was steeper for girls coming from high income municipalities than for girls coming from low income municipalities.

## Supplementary Material

cky235_Supplementary_Data

## Appendix 1 References for tables 1, 2 and 3

A1. INDKP106: Disponible indkomst efter område, enhed, køn, alder og indkomstinterval: Danmarks Statistik; [cited 2017 Jul 04]. Available at: http://www.statistikbanken.dk/INDKP106.

A2. Ingen tilskud til kræftvaccine. Ritzau. 2006 Dec 08 [in Danish].

A3. Kræftvaccine for de velstillede. Århus Stiftstidende. 2007 Feb 08 [in Danish].

A4. Nielsen HF. Både for og imod kræftvaccination. Jyllands-Posten Øst. 2007 May 02 [in Danish].

A5. Gratis kræftvaccine til unge piger. Dagbladet Køge. 2007 May 03 [in Danish].

A6. Kvinder betaler selv kræftvaccine. Politiken. 2007 Aug 09 [in Danish].

A7. Willumsen G. Vaccine mod kræft udløser etisk dilemma. Ingeniøren. 2007 Aug 10 [in Danish].

A8. Kræftvaccine hitter i rige kommuner. Berlingske. 2007 Nov 02 [in Danish].

A9. Kræftvaccine sælger godt i Furesø. Furesø Weekend. 2007 Nov 09 [in Danish].

A10. Hansen JB. Mest udsat gruppe må selv betale vaccine. Århus Stiftstidende. 2007 Nov 12 [in Danish].

A11. Vasbo C. Kræftvaccine sælger bedst i Hørsholm og Rudersdal. Ugebladet Hørsholm. 2007 Nov 21 [in Danish].

A12. Brugerbetaling holder kvinder fra kræftvaccine. Vejle Amts Folkeblad. 2007 Dec 20 [in Danish].

A13. Vaccine mod kræft trækker ud. Fyens Stiftstidende. 2008 Jan 30 [in Danish].

A14. Espersen S. Nordsjælland køber sig fra kræft - Lolland-Falster dropper vaccine. Lolland-Falsters Folketidende. 2008 Nov 22 [in Danish].

A15. Citathistorie fra Fr. borg amts avis: Kræftvaccine med social slagside. Ritzau. 2009 May 03 [in Danish].

A16. Haukrogh LL. Unge kvinder køber vaccine mod livmoderhalskræft. Jyllands-Posten Århus. 2009 May 28 [in Danish].

A17. Birkelund M. Få for himlens skyld vaccinen. Fyens Stiftstidende. 2009 May 06 [in Danish].

A18. Statens Serum Institut. Human papillomavirus-vaccine (HPV) færdigvaccineret - vaccinationstilslutning. Smitteberedskab - Overvågning i tal, grafer og kort [cited 2017 Jul 03]. Available at: http://www.ssi.dk/Smitteberedskab/Sygdomsovervaagning.aspx.

A19. Statens Serum Institut. Human papillomavirus-vaccine (HPV) 1 - vaccinationstilslutningen. Smitteberedskab - Overvågning i tal, grafer og kort [cited 2017 Jul 03]. Available at: http://www.ssi.dk/Smitteberedskab/Sygdomsovervaagning.aspx.
