# Supplementary material for: Stronger responders—uptake and decline of HPV-vaccination in Denmark
Source: Eur J Public Health. 2018 Nov 8;29(3):500–5. doi: 10.1093/eurpub/cky235 (PMC6532829; doi:10.1093/eurpub/cky235)
Supplement: cky235_Supplementary_Data [file cky235_supplementary_data.docx]

**Supplementary Material**

Table 1 Data extracted from newspaper articles from 2006 to 2009 on stronger responders to secure the HPV-vaccine. Average disposable incomes in the municipalities included (S1).

| DD/MM/YY | Source | Total vaccinated | Regions | High income municipalities [income]^1^ | Low income municipalities [income]^1^ | Reference |
| --- | --- | --- | --- | --- | --- | --- |
| 08/12/06 | Danish Drug Information |  |  | 33% sold in North Zealand’s rich municipalities such as Gentofte [300], Holte [285]^2^, Nærum [285]^2^ and Vedbæk [285]^2^ | Not a single dose sold in Ishøj [161] | (S2) |
| 08/02/07 | Sanofi Pasteur | 4,300 |  | Almost 50% sold in area of the capital city, especially in Gentofte [302], Lyngby-Taarbæk [231] and Rudersdal [287] | Almost no one vaccinated in Rødovre [176], Brøndby [166] and Albertslund [165] | (S3) |
| 08/02/07 | Sanofi Pasteur |  | The vaccine is sold twice as much in the Capital region as in the remaining regions |  |  | (S4) |
| 02/05/07 | According to the minister |  |  | The vaccine is more common north of Copenhagen | Not many in Albertslund [165] are vaccinated | (S5) |
| 03/05/07 | Sanofi Pasteur |  | The vaccine is especially being sold in the Capital Region | Sold to women in wealthier municipalities in North Zealand such as Gentofte [302], Lyngby-Taarbæk [231] and Rudersdal [287] | In Brøndby [166] and Albertslund [165] almost no one are vaccinated | (S6) |
| 09/08/07 |  | 12,000 |  |  |  | (S7) |
| 10/08/07 | Sanofi Pasteur |  |  | Mainly girls from the municipalities of North Zealand that are vaccinated | Only very few girls from Rødovre [176], Albertslund [165] and Brøndby [166] are vaccinated | (S8) |
| 06/10/07 |  | 12,000 |  |  |  | (S9) |
| 02/11/07 | Sanofi Pasteur |  | The interest for the vaccine falls the further south and west of Copenhagen you go | 1057 of women aged 16-26 have been vaccinated in Rudersdal [287] and 357 in Helsingør [198] | 57 of women aged 16-26 have been vaccinated in Albertslund [165] | (S10) |
| 09/11/07 | Sanofi Pasteur |  | The interest for the vaccine falls the further south and west of Copenhagen you go | 386 vaccinated in Furesø [227] and 1057 in Rudersdal [287] | 71 vaccinated in Høje Taastrup [180]^3^ and 57 in Albertslund [165] | (S11) |
| 12/11/07 | Sanofi Pasteur | 11,500 | Few vaccinated in Central Denmark Region | Many more vaccinated in the rich municipalities of North Zealand |  | (S12) |
| 21/11/07 |  |  |  | Over 30% of women aged 16-26 vaccinated in Hørsholm [291] and Rudersdal [287] | Of women aged 16-26 3% vaccinated in Høje Taastrup [180]^3^ and 6% in Albertslund [165] | (S13) |
| 20/12/07 |  |  | Per 100,000 citizens:  - 618 doses in the Capital Region  - 527 doses in Region Zealand  - 284 doses in Region of Southern Denmark  - 261 doses in North Denmark Region  - 260 doses in Central Denmark Region |  |  | (S14) |
| 20/12/07 |  |  |  | Per 100,000 citizens in Rudersdal [287] almost 2,000 have been vaccinated | Per 100,000 citizens 180 have been vaccinated in Vejle [180]^3^ and 69 in Horsens [171] | (S15) |
| 21/12/07 |  |  | Twice as many people from Copenhagen have secured the vaccine compared to Jutland |  |  | (S16) |
| 30/01/08 |  | 21,000 |  |  |  | (S17) |
| 22/11/08 | Sanofi Pasteur |  |  | In Hørsholm [260] 2,980 vaccines were sold per 100,000 citizens in 2007 | On Lolland [151] 218 vaccines were sold per 100,000 citizens in 2007 | (S18) |
| 03/05/09 |  |  |  | 1/3 of women between 17-26 years old have bought the vaccine in Hørsholm [251] | 1/50 of women between 17-26 years old have bought the vaccine Høje Taastrup [184]^3^ and Faaborg [167] | (S19) |
| 28/05/09 | Sanofi Pasteur |  |  |  | Almost 15% of women aged 16-26 from Århus [178] have been vaccinated | (S20) |

All quotes translated from Danish to English

^1^ Disposable income in the given year of the newspaper article, rounded to the nearest thousand DKK.

National average disposable incomes in thousands of DKK in the given years: 2006 [173], 2007 [178], 2008 [176] and 2009 [179] (S1).

^2^ These three municipalities were joined in one municipality called Rudersdal as part of a reform in 2007. On Statistics Denmark one can only select the names of the new municipalities, therefore the disposable income of Rudersdal is used to represent the disposable income in these former three municipalities.

^3^ Average disposable income marginally above national average.

**References for Supplementary Material**

S1. INDKP106: Disponible indkomst efter område, enhed, køn, alder og indkomstinterval: Danmarks Statistik; [cited 2017 Jul 04]. Available from: <http://www.statistikbanken.dk/INDKP106>.

S2. Ingen tilskud til kræftvaccine. Ritzau. 2006 Dec 08 [in Danish].

S3. Kræftvaccine for de velstillede. Århus Stiftstidende. 2007 Feb 08 [in Danish].

S4. Skovmand K, Sølund S. Kræftvaccine: vaccinen er vigtigere end pengene. Politiken. 2007 Feb 08 [in Danish].

S5. Nielsen HF. Både for og imod kræftvaccination. Jyllands-Posten Øst. 2007 May 02 [in Danish].

S6. Gratis kræftvaccine til unge piger. Dagbladet Køge. 2007 May 03 [in Danish].

S7. Kvinder betaler selv kræftvaccine. Politiken. 2007 Aug 09 [in Danish].

S8. Willumsen G. Vaccine mod kræft udløser etisk dilemma. Ingeniøren. 2007 Aug 10 [in Danish].

S9. Hjortdal M. Vaccine kan redde 100 liv om året. Politiken. 2007 Oct 06 [in Danish].

S10. Kræftvaccine hitter i rige kommuner. Berlingske. 2007 Nov 02 [in Danish].

S11. Kræftvaccine sælger godt i Furesø. Furesø Weekend. 2007 Nov 09 [in Danish].

S12. Hansen JB. Mest udsat gruppe må selv betale vaccine. Århus Stiftstidende. 2007 Nov 12 [in Danish].

S13. Vasbo C. Kræftvaccine sælger bedst i Hørsholm og Rudersdal. Ugebladet Hørsholm. 2007 Nov 21 [in Danish].

S14. Færrest jyske piger vaccineres. Vejle Amts Folkeblad. 2007 Dec 20 [in Danish].

S15. Brugerbetaling holder kvinder fra kræftvaccine. Vejle Amts Folkeblad. 2007 Dec 20 [in Danish].

S16. Korsbæk M. Livmoderhalskræft: Gratis kræftvaccine med i finanslov. Fredericia Dagblad. 2007 Dec 21 [in Danish].

S17. Vaccine mod kræft trækker ud. Fyens Stiftstidende. 2008 Jan 30 [in Danish].

S18. Espersen S. Nordsjælland køber sig fra kræft - Lolland-Falster dropper vaccine. Lolland-Falsters Folketidende. 2008 Nov 22 [in Danish].

S19. Citathistorie fra Fr. borg amts avis: Kræftvaccine med social slagside. Ritzau. 2009 May 03 [in Danish].

S20. Haukrogh LL. Unge kvinder køber vaccine mod livmoderhalskræft. Jyllands-Posten Århus. 2009 May 28 [in Danish].
